# Multi-Scale Oriented Detection with Shared Convolution for UAV-Enabled Maritime Safety Surveillance

**DOI:** 10.34133/research.0920

**Published:** 2025-10-07

**Authors:** Yanhong Huang, Yijie Zheng, Peng Wu, Yao Zhang, Jingxian Liu, Yuanchang Liu

**Affiliations:** ^1^ School of Navigation, Wuhan University of Technology, Wuhan, China.; ^2^ Department of Mechanical Engineering, University College London, London, UK.; ^3^ School of Control Science and Engineering, Dalian University of Technology, Dalian, China.

## Abstract

Due to their ability to cover wide areas and adapt to variable perspectives, unmanned aerial vehicles (UAVs) equipped with high-definition cameras have become effective devices for maritime safety management. However, the changing visual angles, varying flight distances, and limited computational power pose challenges for maritime safety surveillance using UAVs. These challenges often result in inaccurate multi-angle detection, rough multi-scale vessel detection, and computational strain from large models. Therefore, we propose a lightweight multi-scale oriented detection model for UAVs. Specifically, to accommodate variable flight altitudes, we firstly proposed a cross-stage partial feature fusion module named LDFusion, which can freely adjust the size and shape of the convolutional kernel to extract and fuse features at different scales. While the LDFusion module improves feature extraction performance, it also introduces additional parameters. Therefore, we secondly designed a lightweight detection head with shared convolution module SConvs for oriented ship detection, reducing the number of parameters. Thirdly, we created 3 oriented datasets from a maritime UAV perspective, including a new inland waterway dataset, a re-annotated marine dataset, and a re-annotated complex maritime dataset. Finally, we conducted comparative experiments on the 3 datasets using advanced oriented detection methods. Experimental results demonstrate that though our method achieves a modest 3.27% improvement in detection accuracy, it reduces the number of parameters by 24.40% compared to the latest approach.

## Introduction

**Fig. 1.**
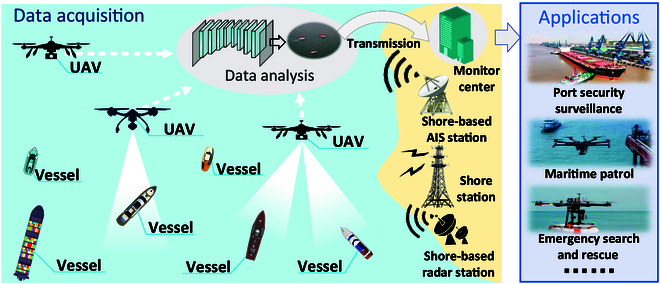
The flowchart of maritime safety surveillance system with UAVs, which includes data acquisition, data analysis, and maritime surveillance applications. The data acquisition mainly includes visual data with UAVs from different sources.

The acceleration of globalization and marine resource development has posed unprecedented challenges to maritime safety management [1]. Maritime management encompasses a wide range of activities, including environmental surveillance, illegal fishing, and smuggling activities regulation. However, the vastness and complexity of the waterway environment bring serious limitations to traditional surveillance methods. Although traditional methods (vessel patrols, radar monitoring, and satellite remote sensing) can provide a certain level of surveillance, they suffer from high costs and poor real-time capabilities, limited coverage, and insufficient accuracy. With the rapid development of internet of things (IoT) technology, various fields are gradually moving toward digitalization and intelligence. As efficient IoT nodes, unmanned aerial vehicles (UAVs) are widely used in maritime safety management for environmental perception and surveillance [2]. In particular, UAVs are currently equipped with various sensors to conduct safety surveillance in dangerous areas where traditional infrastructure is insufficient and absent [3] and difficult for humans to reach. In addition, UAVs offer advantages of high flexibility, easy operation, low cost, wide coverage, and high data collection accuracy, showing great potential in maritime safety surveillance (as shown in Fig. 1). Due to the broad perspective, economic cost, and high surveillance efficiency, camera-equipped UAVs have become popular in maritime safety surveillance. They aim to accurately detect and identify objects captured in visual images.

**Fig. 2.**
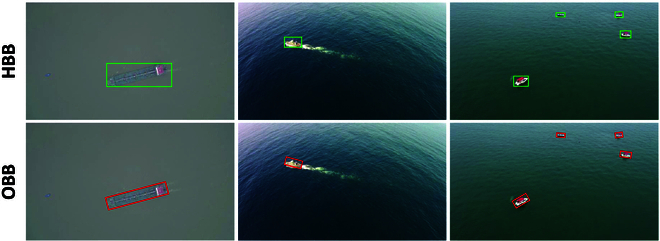
Comparison of annotation methods between horizontal and oriented bounding boxes. The first row is annotated with horizontal bounding boxes, while the second row is tightly annotated with oriented bounding boxes.

With the rapid development of machine learning, deep learning-based object detection methods have substantially improved in real-time performance and accuracy. Recently, faster region-based convolutional neural network (faster R-CNN) [4], you only look once (YOLO) [5–8], and other methods have been applied to vessel detection tasks. Although these networks can achieve satisfactory detection accuracy, they involve a large number of parameters, making them overly complex for vessel detection from a UAV perspective and difficult to meet the real-time requirements of maritime surveillance. Moreover, these methods often fail to account for the multi-scale and multi-angle characteristics of vessel targets. As a result, when applied to UAV-based vessel detection tasks, they not only suffer from overfitting but also prove unsuitable for resource-constrained onboard processing platforms. Additionally, existing datasets, e.g., the Microsoft common objects in context (COCO) dataset [9] and the Pascal visual object classes challenge (VOC) dataset [10], are mostly captured from a single perspective and contain limited maritime-specific data. Although the Seagull dataset [11] and MS2Ship [12] dataset are maritime-specific datasets, they are both annotated with horizontal bounding boxes (HBBs), neglecting the directional differences of targets from the perspective (as shown in Fig. 2). The variations in UAV flight altitude and orientation pose serious challenges to the accurate recognition of multi-angle and multi-scale vessels, adversely affecting detection and tracking performance from the UAV perspective.

### Traditional maritime safety surveillance

Traditional vessel dynamic monitoring mainly relies on the automatic identification system (AIS) [13] and radar [14]. These methods excel in vessel positioning and navigation monitoring, particularly radar, which provides reliable monitoring under adverse weather conditions and long-range scenes. However, the vessel information is actively sent by AIS, which can be intentionally tampered with, turned off, or disrupted. It decreases the reliability of AIS data, leading to an inability to comprehensively monitor all vessels. Additionally, while radar has a wide detection range, it fails to provide high-resolution images of vessels, making it difficult to distinguish between different vessel types and identify small targets. In contrast, due to their cost-effectiveness and ease of deployment, cameras are increasingly playing an important role in maritime safety surveillance. With the development of the visual detection method, cameras can not only provide intuitive image data but also perform object detection and tracking. Currently, deep learning-based visual surveillance methods have become the mainstream in maritime safety surveillance. Substantial efforts have been made to exploit efficient detection methods. Compared to 2-stage detection methods [4], single-stage methods complete detection with a single net. These models with simpler network structures and lower computational requirements can better meet industrial needs. MobileNet [15] is a typical single-stage object detection model. MobileNet [15] employs depth-wise separable convolution to build a lightweight network, reducing the parameters and computational demands. Additionally, the feature pyramid network (FPN) [16] combines features from multiple scales, enabling semantic extraction across different levels. Obviously, the one-stage detection models based on YOLO [5–8] have rapidly and overwhelmingly adapted to various industries. YOLOv1 [5] is the first to transform the object detection task into a regression. It exploits a convolutional neural network (CNN) to directly generate bounding boxes and class probabilities from images. YOLOv3 [6] further makes the networks lightweight. Moreover, YOLOv5 [7] designs the lightweight network by introducing cross stage partial networks (CSPNet) to avoid redundant calculations. Recently, YOLOv8 [8] provides the cross stage partial feature fusion module C2F and maintains fast inference speeds.

Traditional surveillance methods have both advantages and disadvantages (as shown in Table 1). Considering the economic and flexibility factors, the role of cameras in surveillance should not be overlooked. Methods for processing images have attracted increasing attention. Although many approaches can automatically extract features from large datasets to improve the accuracy of vessel detection and dynamic surveillance, the equipment mounted with cameras can affect the quality of the collected data. Compared to the flexibility and real-time detection capabilities of UAVs, despite these methods showing good detection performance in maritime scenarios, shore-based visual surveillance is limited by its coverage range and cannot surveil distant sea areas. Its effectiveness markedly decreases beyond its field of view. Although remote sensing satellites can cover large areas, their data acquisition frequency is low, making real-time dynamic surveillance difficult. Moreover, remote sensing is costly and can be hindered by cloud cover. Therefore, cameras mounted on UAVs play an indispensable role in maritime surveillance.

**Table 1.** Advantages and disadvantages of the main equipment for maritime surveillance

Equipment	Advantages	Disadvantages
AIS	Provide dynamic/static information of vessels	Low reliability Data noise
Radar	Long range All weather	Affected by sea clutter High cost
Camera	Low cost High resolution	Affected by weather Short range

### UAV-assisted maritime safety surveillance

UAV-mounted visual detection provides a flexible and cost-effective alternative in maritime safety surveillance. UAV can quickly reach target areas and capture high-resolution images, achieving precise vessel detection and dynamic tracking. Due to flexible deployment and low operating costs, UAV-mounted visual detection becomes an effective means in current maritime vessel dynamic surveillance. It complements traditional measures and improves the overall efficiency of maritime safety surveillance. However, objects are typically oriented in irregular directions from an aerial perspective. Since vessels in UAV views appear at different angles, conventional HBB results [17] are in large background areas. Therefore, oriented object detection methods are more suitable for UAV-view data. The rotating box detection can adjust the bounding box direction according to the actual target posture and frame the targets more correctly. Oriented bounding box (OBB) detection was used for tilted text detection in the early stage [18] and then gradually applied to aerial image detection. Based on faster R-CNN [4], Xie et al. [19] propose oriented R-CNN. They introduce an oriented region proposal network that generates high-quality oriented proposals with nearly no extra cost. Unlike 2-stage detection, Zhang et al. [20] propose an improved YOLO. As a single-stage OBB method, it improved the speed of OBB based on YOLO. They adjust the position of rotated anchors to achieve a more accurate regression. In the maritime field, Liu et al. [21] employ rotation region CNN to extract the features of the vessel and accurately locate rotating objects.

Single-stage object detection methods generally offer faster inference speeds because they combine object localization and classification into a unified network process, reducing computational overhead. Compared to 2-stage methods [4,19,22–25], single-stage methods [8,26–30] are more suitable for scenarios with high real-time requirements. However, in dynamic maritime surveillance tasks carried out by UAVs, the devices themselves are edge terminals with limited computational resources, necessitating a careful balance between inference speed, detection accuracy, and hardware constraints. As a fundamental task in maritime surveillance, visual object detection often requires collaboration with other complex tasks, such as object tracking, multi-sensor fusion, and anomaly detection, which further increases demands on model efficiency. Although large detection models achieve excellent accuracy, their substantial computational load and memory requirements make them unsuitable for deployment on resource-constrained UAV platforms, limiting their real-time applicability and practical value. Moreover, the maritime environment presents substantial detection challenges due to the wide range of object scales, diverse orientations, and high spatial complexity. Lightweight OBB detection models are computationally more efficient but face considerable difficulty in maintaining high detection accuracy while remaining lightweight, especially for fine-grained recognition of multi-scale and multi-angle targets. This trade-off between accuracy and efficiency is a key challenge that UAV-based maritime visual detection technology must overcome.

### Motivations and contributions

Deep learning has achieved remarkable success in various object detection tasks due to its powerful capabilities in feature extraction and abstraction. However, UAV-assisted surveillance in maritime scenes still faces several critical challenges. First, the deployment of large-scale models imposes major computational burdens on UAVs [31], which are typically edge devices with limited processing capabilities. Second, existing object detection algorithms struggle to adapt to the complexities of maritime traffic scenes. Compared to urban traffic environments [32–34], maritime settings exhibit higher background variability and uncertainty, with greater diversity in the size and appearance of vessel targets. In particular, maritime UAV surveillance faces unique challenges due to the highly dynamic water surface textures and the wide variation in vessel types, scales, and orientations. Current object detection approaches still lack sufficient adaptability in such aquatic environments. Finally, despite the abundance of general-purpose object detection datasets, there remains a scarcity of datasets specifically designed for oriented vessel detection on water surfaces. To address these issues, we first construct 3 maritime datasets from a UAV perspective that capture a wide range of real-world scenes. Based on these datasets, we further develop a lightweight and stable oriented vessel detection model tailored to the maritime domain, aiming to enhance the real-time surveillance capabilities of regulatory authorities. The main contributions of this paper are as follows:

• A cross-stage partial feature fusion module (termed LDFusion) is proposed to freely fuse the features with different scales. It employs linear deformable convolution to extract features at different scales and fuses them. LDFusion is provided to not only learn more ship details but also avoid the quadratic growth of the parameters caused by the fixed square matrix convolution kernel.
• An oriented object detection head (termed Shared Head) with the novel shared convolution module (termed SConvs) is constructed to adjust the bounding box directions according to the actual posture of objects. It addresses the challenge of accurately framing tilted or rotated objects using HBBs. At the same time, the SConvs module reduces the model parameters in the head.
• Three maritime datasets with oriented annotations are constructed to validate the effectiveness of the proposed method. The InlandVessel dataset is collected from the Yangtze River. Additionally, the SeagullOBB dataset and the MS2ShipOBB dataset are re-annotated using OBBs from Seagull [11] and MS2Ship [12], respectively.
• Comparative experiments are conducted on the inland dataset and the marine dataset and complex maritime dataset by using advanced OBB detection methods. The experimental results indicate that, compared to the latest method, though our approach achieves a modest accuracy improvement of 3.27%, it greatly reduces the number of parameters by 24.40%.

## Results and Discussion

To verify the effectiveness and superiority of the proposed method, we conduct a series of experiments on 3 datasets. This section introduces the performance metrics, experimental datasets, experimental environment, and training details. Further experimental comparative analysis is conducted on inland waterway scenes, marine scenes, and complex maritime scenes datasets. Moreover, the ablation study proves the effectiveness of the proposed module.

### Performance metrics

To verify the reliability and accuracy of the target detection algorithm, we usually use some quantitative evaluation indicators to judge whether the algorithm can meet the actual application requirements. Common target detection evaluation indicators are mainly the following, i.e., recall (R), precision (P), and mean average precision (mAP).

#### Recall and precision

Recall (R) and precision (P) are key indicators for evaluating the performance of target detection model. R and P represent the ability of the evaluation model to identify true-positive samples, which respectively represent the proportion of correctly detected positive samples to all true-positive samples and the proportion of correctly detected positive samples to the number of detected positive samples, reflecting the ability to capture true-positive samples.

$$\text{Recall}=\frac{\mathrm{TP}}{\mathrm{TP}+\mathrm{FN}}$$ (1)

$$\text{Precision}=\frac{\mathrm{TP}}{\mathrm{TP}+\mathrm{FP}}$$ (2)

where true positive (TP) denotes the number of correctly predicted positive samples, false negative (FN) refers to the number of actual positive samples misclassified as negative, and false positive (FP) indicates the number of negative samples incorrectly predicted as positive.

#### Mean average precision

mAP is a widely used evaluation criterion in the field of object detection, which is used to comprehensively measure the robustness and accuracy of the detection model.

$$\mathrm{AP}=\int_{0}^{1}P\left(R\right)\mathrm{dR}$$ (3)

$$\mathrm{mAP}=\frac{1}{N}\sum_{i=1}^{N}{\mathrm{AP}}_{n}$$ (4)

with AP50 being the average precision when the intersection over union (IoU) threshold is 0.5. A higher mAP value usually means that the target detection model can provide more accurate and more effective results in the vessel detection task. The comprehensive evaluation of these 3 indicators can more comprehensively reflect the performance of the target detection network.

### Experimental datasets

Despite the existence of numerous maritime datasets (Seagull dataset [11], MS2Ship dataset [12], AFO dataset [35], SeaDronesSee dataset [36], MOBDrone dataset [37], etc.), they primarily use HBBs for annotation. However, since targets obtained from a UAV perspective have orientation, we construct 3 oriented detection maritime datasets. To account for different maritime scenes, we validated the proposed multi-scale lightweight detection method on 3 real-world datasets. Specifically, the experimental datasets cover 3 distinct scenes, i.e., InlandVessel containing only inland waterway scenes, SeagullB covering only marine scenes, and MS2ShipOBB comprising a variety of maritime environments. These datasets are designed to better capture the directional characteristics of targets, particularly in dynamic UAV-mounted maritime safety surveillance, providing more accurate detection results.

#### InlandVessel dataset

**Fig. 3.**
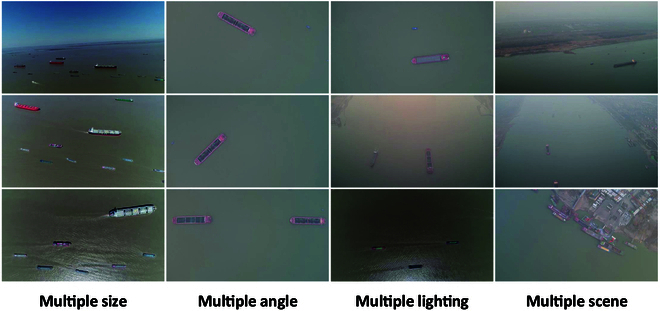
Visual presentation of the InlandVessel dataset. It includes multi-size vessels, multi-angle targets, multi-light conditions, and multi-scene inland scenes.

Inland waterways serve as the primary channels for vessel navigation. However, inland surveillance often encounters complex conditions involving vessels of various sizes and orientations, diverse lighting conditions, and a wide range of inland water scenes. To evaluate detection performance under such challenging conditions, we collect over 10 videos of the Yangtze River in the Wuhan section using UAVs. The videos are then processed through frame extraction and filtering, primarily to remove frames without vessel targets. A large number of images containing multi-scale vessels are annotated, reflecting the varying target scales caused by UAVs flying at different altitudes. We use roLabelImg to provide OBB annotations, taking into account the dynamic changes in viewing angles during UAV-based maritime surveillance. As a result, we construct the InlandVessel dataset (as shown in Fig. 3), which contains 5,746 inland maritime images with a resolution of 3,840 × 2,160. This dataset provides a solid foundation for multi-scale vessel detection, particularly for tasks involving target detection and dynamic surveillance in complex inland waterway environments.

#### SeagullOBB dataset

**Fig. 4.**
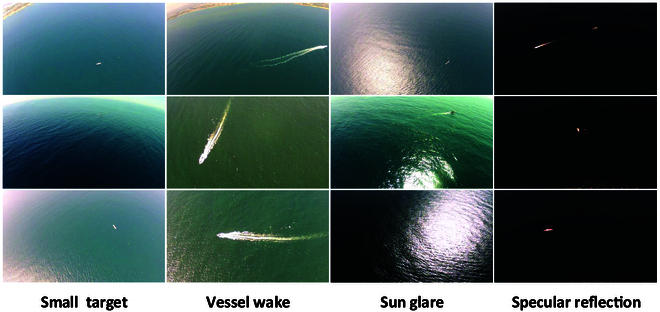
Visual presentation of the SeagullOBB dataset. It is composed of rich maritime scenes. From left to right are images with small target, vessel wake, sun glare, and specular reflection, respectively.

Maritime surveillance poses substantial challenges due to the vastness of the ocean and complex environmental factors such as small targets, vessel wakes, sun glare, and specular reflections. UAVs have proven to be effective tools for such tasks, but these challenges can substantially impact detection performance. To address these issues, we conduct targeted experiments based on the Seagull dataset [11] to evaluate the generalization of the proposed model in real-world maritime scenarios. The Seagull dataset contains 6,666 images and supports a variety of maritime tasks. However, it employs HBBs for annotation, which inadequately capture the orientation of vessel targets. To overcome this limitation, we re-annotate the dataset using OBBs to better encode directional information. We also filter out images without vessels and provide refined annotations for all remaining targets. The resulting dataset, named SeagullOBB (as shown in Fig. 4), includes images of varying resolutions (1,920 × 1,080, 1,024 × 768, 640 × 480, 384 × 288, and 1,024 × 648 pixels). These OBB annotations enable more accurate orientation-aware detection and markedly enhance the performance of our model in multi-angle and real-time UAV surveillance scenarios.

#### MS2ShipOBB dataset

**Fig. 5.**
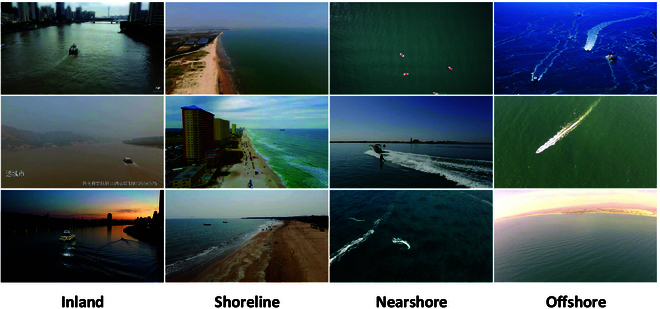
Visual presentation of the MS2ShipOBB dataset. It is divided into 4 scenes, i.e., inland, shoreline, nearshore, and offshore.

The MS2Ship dataset [12] covers a wide range of complex waterborne traffic scenarios (e.g., inland, shoreline, nearshore, and offshore), offering a comprehensive benchmark for model evaluation. It comprises 6,470 images with varying resolutions and aspect ratios, ensuring diversity and comprehensiveness, and serving as a robust foundation for maritime target detection using HBBs. However, despite its extensive coverage, MS2Ship lacks the capability to accurately represent vessel orientation in multi-angle UAV surveillance scenarios. To address this limitation, we re-annotate MS2Ship with OBBs, resulting in the MS2ShipOBB dataset (as shown in Fig. 5). This dataset includes vessels of various scales annotated with orientation information, and incorporates challenging scenarios such as water surface reflections and low-light conditions. It is better suited for maritime UAV surveillance tasks, offering improved accuracy in both target detection and orientation estimation. Additionally, the maritime scenario is inherently complex, as it involves not only vessels but also various obstacles, particularly in shoreline and nearshore scenarios, and there are various human activities. To evaluate the comprehensiveness of MODet in maritime surveillance, we re-annotated the SeaDronesSee [36] dataset to construct a new maritime OBB dataset encompassing 3 classes, i.e., vessel, human, and obstacle. Furthermore, we utilized the MS2ShipOBB dataset as a pretraining dataset to further enhance MODet.

### Experimental settings

The training of our MODet was conducted on a PC powered by a 12th Gen Intel(R) Core (TM) i5-12400F processor with an NVIDIA GeForce RTX 3060 Ti GPU. MODet was implemented using the PyTorch 1.12.1 package on the Python 3.10 platform. Moreover, the AdamW optimizer was employed for model training, with the momentum set to 0.9. Training was conducted for 100 epochs, with an initial learning rate of 0.002 and a batch size of 8. The loss function weights were set as $\omega_{1}=7.5$, $\omega_{2}=0.5$, and $\omega_{3}=1.5$. To expedite convergence, the COCO dataset [9] was exploited for pretraining. For fair comparisons, the hyperparameters of competing methods, including Faster R-CNN OBB [24], Oriented R-CNN [38], RetinaNet OBB [28], Double Head OBB [23], FCOS OBB [27], Gliding Vertex [25], RoI Transformer [22], S2A-Net [26], YOLOv8 OBB [8], YOLOv11 OBB [29], and YOLOv12 OBB [30], were configured according to their optimal settings. Given the limited number of the 3 datasets, we perform *K*-fold cross-validation (*K* = 5) to ensure a more robust evaluation of the performance. Additionally, considering the resource limitations of UAV platforms, we conduct inference on a PC equipped with an NVIDIA GeForce GTX 1060 GPU (6 GB) and report the frames per second (FPS) and memory usage to highlight its practicality for real-world UAV deployment.

### Experimental results on inland waterway scenes

**Fig. 6.**
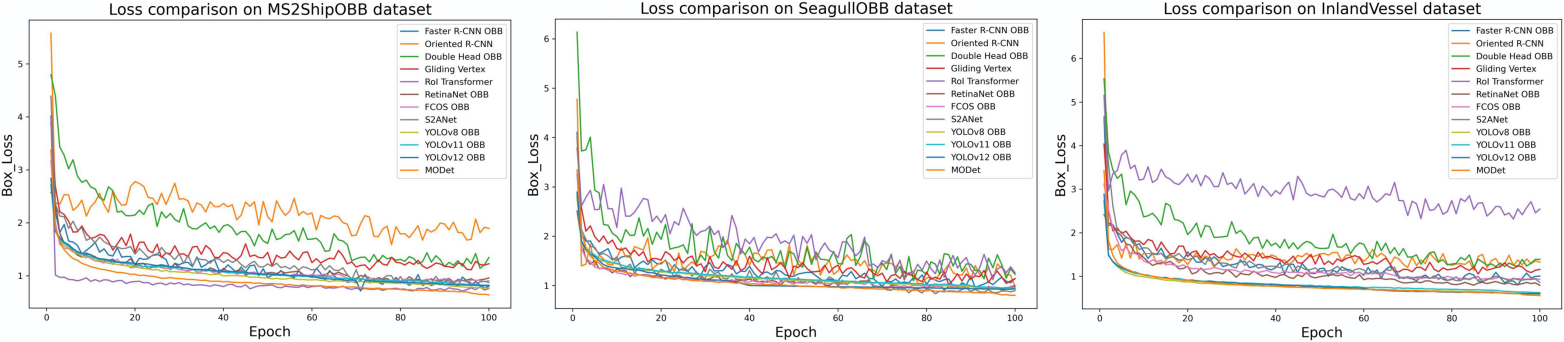
Loss comparison on 3 datasets. It includes the loss results of 12 methods across inland waterway scenes, marine scenes, and complex maritime environments. The results indicate that MODet achieves smoother and lower loss on all 3 datasets.

The quantitative experimental results of all 12 methods are shown in Table 2. The visualization results of their losses are shown in Fig. 6. Since our dataset contains only one class, the AP50 and mAP50 values are the same; therefore, only the AP50 results are presented. As the most classic 2-stage detection method, Faster R-CNN OBB [24] uses ResNet as its backbone and achieves the lowest recall and accuracy. Double Head OBB [23], due to its dual-head design, has more parameters. The increased model complexity slows down the inference speed, though it contributes to improved detection performance. Compared to traditional 2-stage detectors, RoI Transformer [22] introduces a transformer module to replace CNN for extracting region proposal features. Although it captures more complex feature space information, it requires more computational resources, resulting in the largest number of parameters and suboptimal inference speed. Although S2ANet [26] is an end-to-end detection method, its complex spatial sequence attention mechanism leads to a relatively high parameter count and slow inference speed. In contrast, the anchor-free design of FCOS OBB [27] substantially improves both detection accuracy and inference speed. However, it still lags behind YOLO-based methods [8,29,30]. YOLO-based methods [8,29,30] adopt CSPDarknet for lightweight feature extraction, reducing redundant parameters while maintaining high detection accuracy. Furthermore, our proposed MODet, built upon the YOLO framework, incorporates LDFusion and SConvs modules to avoid the exponential parameter growth caused by square convolution, while achieving the highest detection accuracy.

**Table 2.** Quantitative comparisons on 3 UAV maritime datasets. The experimental results are obtained using *K*-fold cross-validation with K=5, and the average performance is reported. The best performance is highlighted in bold, while the second-best results under the same parameters are indicated by underline. The FPS and memory usage metrics represent the frames per second and GPU memory in inference, respectively.

Methods	Model/M	InlandVessel				SeagullOBB				MS2ShipOBB			
		AR	AP50	FPS	Memory	AR	AP50	FPS	Memory	AR	AP50	FPS	Memory
Faster R-CNN OBB [24]	314.96	0.8853	0.7912	6.22	3.95G	0.8782	0.8081	6.24	3.93G	0.8235	0.7721	6.62	3.82G
Oriented R-CNN [38]	315.08	0.8991	0.8645	6.14	4.23G	0.8810	0.8105	6.68	4.12G	0.8176	0.7872	7.58	4.10G
Double Head OBB [23]	366.29	0.9202	0.8817	5.58	4.12G	0.8441	0.7993	5.88	3.98G	0.8579	0.8013	6.73	3.98G
Gliding Vertex [25]	314.99	0.8796	0.8058	6.18	4.51G	0.8812	0.8086	6.77	4.42G	0.8891	0.8011	7.61	4.41G
RoI Transformer [22]	421.11	0.9405	0.9011	6.02	4.68G	0.9032	0.8582	6.42	4.63G	0.8486	0.8055	8.28	4.61G
RetinaNet OBB [28]	276.83	0.9203	0.8797	6.98	3.62G	0.9225	0.8842	7.24	3.59G	0.8742	0.8063	8.92	3.60G
FCOS OBB [27]	244.32	0.9592	0.8856	7.58	3.25G	0.9291	0.8893	7.68	3.12G	0.8879	0.8048	9.13	3.11G
S2ANet [26]	277.26	0.9627	0.9051	8.26	3.61G	0.9197	0.8911	8.68	3.58G	0.8773	0.8064	9.81	3.57G
YOLOv8 OBB [8]	6.14	0.9148	0.9513	40.36	2.34G	0.9221	0.9576	40.27	2.33G	0.9024	0.9517	40.55	2.32G
YOLOv11 OBB [29]	5.42	0.9088	0.9537	**41.02**	1.44G	0.9092	0.9616	**41.31**	1.31G	0.8643	0.9242	**41.35**	1.29G
YOLOv12 OBB [30]	5.45	0.9342	0.9541	40.53	1.86G	0.9275	0.9642	40.82	1.82G	0.9022	0.9511	41.16	1.82G
MODet (ours)	**4.12**	**0.9665**	**0.9603**	40.67	**1.24G**	**0.9493**	**0.9722**	41.07	**1.19G**	**0.9551**	**0.9822**	41.32	**1.18G**

Across the 3 different datasets, one-stage detection models generally exhibit smaller model sizes, faster inference speeds, and lower memory usage, making them more suitable for real-time applications. Although 2-stage models tend to have more parameters, their performance advantage is only observed in the simpler InlandVessel dataset. In contrast, they yield the lowest detection accuracy in the more complex maritime scenarios. YOLO-based [8,29,30] methods demonstrate stronger capabilities in handling multi-angle detection under complex conditions. Additionally, we further validated the model performance across different object scales, as shown in Table 3. Since vessels in inland scenes are typically larger in size, all 12 methods achieved higher detection accuracy on large-scale vessel targets. Among them, YOLO-based approaches delivered high-precision results across all stages. For the SeagullOBB dataset, which contains complex disturbances such as vessel wakes and intense reflections, our proposed MODet outperforms the latest YOLOv12 [30] by leveraging linearly deformable convolutional kernels to better capture fine-grained details of vessel targets, thereby enhancing the robustness under challenging viewing angles. On the more complex MS2ShipOBB dataset, MODet achieves consistently strong performance across small, medium, and large targets, due to the superior capability of LDFusion in extracting and fusing multi-scale features.

**Table 3.** The mAP values of 12 methods for small-, medium-, and large-scale objects on 3 datasets. The best performance is highlighted in bold, while the second-best results under the same parameters are indicated by underline.

Methods	InlandVessel			SeagullOBB			MS2ShipOBB		
	Small	Medium	Large	Small	Medium	Large	Small	Medium	Large
Faster R-CNN OBB [24]	0.6133	0.6785	0.8183	0.7526	0.7901	0.8521	0.7602	0.7513	0.8452
Oriented R-CNN [38]	0.7491	0.8113	0.8958	0.8312	0.8690	0.9032	0.8303	0.8437	0.8679
Double Head OBB [23]	0.7907	0.8493	0.9165	0.8508	0.8878	0.9090	0.8439	0.8762	0.8905
Gliding Vertex [25]	0.6558	0.7020	0.8363	0.7706	0.8081	0.8621	0.7548	0.7967	0.8476
RoI Transformer [22]	0.7843	0.8212	0.9362	0.8677	0.9071	0.9415	0.8572	0.8896	0.9217
RetinaNet OBB [28]	0.7902	0.8450	0.9131	0.8468	0.8851	0.9062	0.8275	0.8713	0.8976
FCOS OBB [27]	0.7970	0.8521	0.9204	0.8525	0.8921	0.9136	0.8436	0.8879	0.9097
S2ANet [26]	0.7796	0.8512	0.9405	0.8703	0.9102	0.9203	0.8409	0.9072	0.9272
YOLOv8 OBB [8]	0.8713	0.885	0.9822	0.9211	0.9547	0.9812	0.9015	0.9357	0.9717
YOLOv11 OBB [29]	0.8779	0.898	0.9850	0.9242	0.9578	0.9815	0.9190	0.9415	0.9786
YOLOv12 OBB [30]	0.8785	0.9011	0.9869	0.9305	0.9588	**0.9821**	0.9216	0.9503	**0.9809**
MODet (ours)	**0.8872**	**0.9320**	**0.9902**	**0.9323**	**0.9650**	0.9818	**0.9243**	**0.9579**	0.9802

**Fig. 7.**
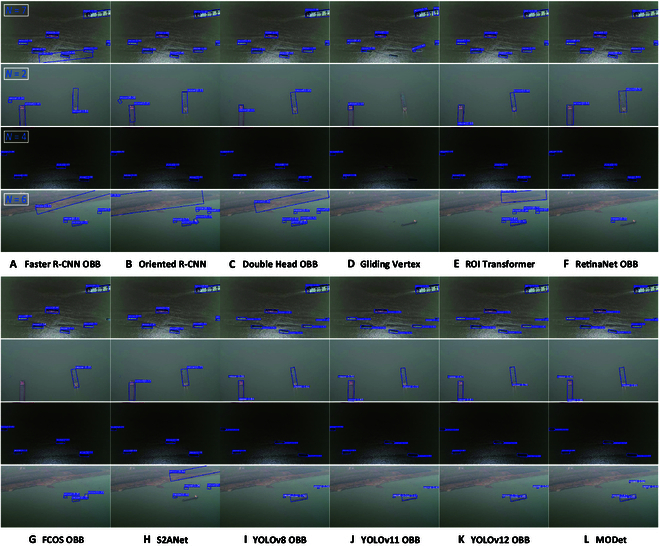
Visual presentation of 12 oriented detection methods on the InlandVessel dataset. Four distinctive images from the InlandVessel dataset are selected as demonstration examples. From the first row to the fourth row, they represent multi-size, multi-angle, multi-illumination, and multi-scene scenarios, respectively. (A) to (L) show the experimental results of 12 methods, i.e., (A) Faster R-CNN OBB, (B) Oriented R-CNN, (C) Double Head OBB, (D) Gliding Vertex, (E) ROI Transformer, (F) RetinaNet OBB, (G) FCOS OBB, (H) S2ANet, (I) YOLOv8 OBB, (J) YOLOv11 OBB, (K) YOLOv12 OBB, and (L) MODet. The total number of annotated targets is displayed in the top-left corner of the first column.

The visualized detection results are shown in Fig. 7. The comparison of visual results demonstrates that in 2-stage detection methods, Gliding Vertex [25] focuses more on the offset of inclined bounding box vertices and the position regression of the box. It makes the dynamic optimization of vertices extremely challenging. Regardless of the size, angle, illumination, or scenario, Gliding Vertex [25] performs the worst in detection results. In contrast, Faster R-CNN OBB [24] benefits from its simpler box regression, which gives it an advantage. Building on Faster R-CNN OBB [24], Oriented R-CNN [38] optimizes the tilted characteristics of the region of interest (ROI), reducing feature errors caused by converting horizontal boxes into rotated ones. Double Head OBB [23] adds a parallel regression head, and its strong feature separation capability gives it superior performance in inclined object detection tasks. Although Oriented R-CNN [38] and Double Head OBB [23] outperform Faster R-CNN OBB [24], their detection accuracy still lags behind RoI Transformer [22], which frequently generates false and missed detections. Furthermore, since RoI Transformer [22] directly affects feature transformation, it is more sensitive to the directional and positional characteristics of small objects, yielding excellent detection results in inland waterway scenarios. However, the computational complexity and inference speed of 2-stage methods limit their deployment on small UAVs. Among one-stage methods, FCOS OBB [27] performs the worst in multi-angle detection, due not only to the detail loss caused by haze in the dataset but also to its lack of angle regression. Based on it, the YOLO-based oriented box detection adopts the probability of IoU (ProbIoU) loss function, which incorporates rotational angle regression, leading to better performance in multi-angle target detection. As shown in Fig. 7, YOLOv12 OBB [30] excels in detecting vessels from multiple angles and multiple scenes. Nevertheless, under challenging lighting conditions and varying object sizes, our method exhibits superior robustness, benefitting from its multi-scale feature extraction design.

### Experimental results on marine scenes

**Fig. 8.**
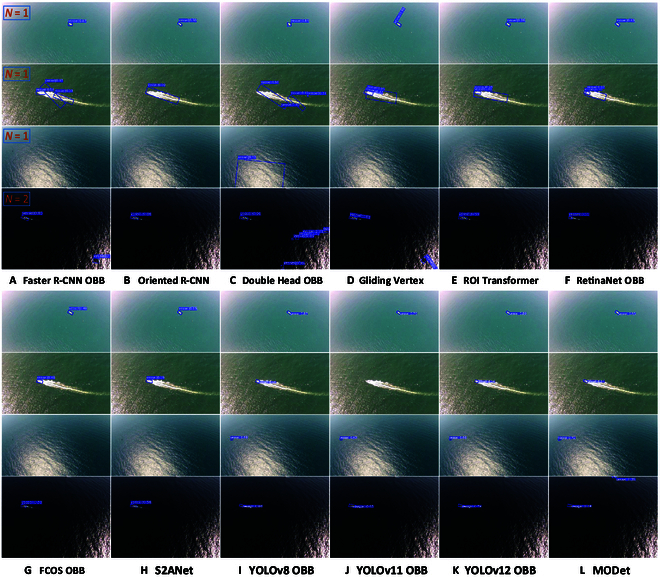
Visual presentation of 12 oriented detection methods on the SeagullOBB dataset. Four distinctive images from the SeagullOBB dataset are selected as demonstration examples. From the first row to the fourth row, they are images with small targets, vessel wakes, sun glare, and specular reflection, respectively. (A) to (L) show the experimental results of 12 methods, i.e., (A) Faster R-CNN OBB, (B) Oriented R-CNN, (C) Double Head OBB, (D) Gliding Vertex, (E) ROI Transformer, (F) RetinaNet OBB, (G) FCOS OBB, (H) S2ANet, (I) YOLOv8 OBB, (J) YOLOv11 OBB, (K) YOLOv12 OBB, and (L) MODet. The total number of annotated targets is displayed in the top-left corner of the first column.

Although MODet performs well on the InlandVessel dataset, InlandVessel only represents inland waterway data and fails to reflect the characteristics of marine scenarios. To evaluate the applicability of MODet in marine environments, we conducted experiments on the SeagullOBB dataset. The SeagullOBB dataset with small targets, vessel wakes, sun glare, and specular reflections provides a comprehensive challenge to test the performance. The visualized detection results on the SeagullOBB dataset are shown in Fig. 8. The comparison of visual results demonstrates that while 2-stage detection methods still excel in small object detection, they frequently misdetect targets in images with vessel wakes. For marine scenarios with glare and specular reflections, both missed detections and false positives occur. Since the sun glare blurs the features of the target and introduces background noise, it limits the performance of the oriented detection methods based on Faster R-CNN [4]. Although RetinaNet OBB [28] eliminates biases generated in region proposal, strong light diminishes the effectiveness of feature extraction, leading to poor detection of small targets under glare. FCOS OBB [27] relies on the center point for target prediction. However, the sun glare causes the features in the central region of targets to become blurred, preventing the model from accurately locating the center point. Additionally, the anchor-free FCOS OBB [27] is highly dependent on target shape. Moreover, in glare scenarios, the blurred boundaries of targets lead to inaccuracies in predicting the target shape and rotation angle. S2ANet [26] with AlignConv aligns features based on anchor size, shape, and orientation. However, glare and reflections cause target edges to disappear, making it difficult to accurately capture anchor shapes and boundaries and finally resulting in reduced detection accuracy. In contrast, the YOLO-based [8,29] oriented detection methods no longer emphasize feature alignment. Their anchor-free mechanism eliminates the anchor generation and refinement steps, directly regressing the center point, size, and rotation angle of the target on the feature map. Therefore, they can reduce the potential for cumulative errors. Besides, in complex scenarios (e.g., sun glare and specular reflections), YOLOv8 [8] rejects relying on anchors to initialize boxes, allowing it to more flexibly adapt to variations in target shape and background. Compared to other YOLO-based methods, we observed that YOLOv12 [30] achieves more accurate detection for standard scenes. However, its performance substantially degrades when facing vessel wakes, sun glare, and specular reflections. In contrast, our MODet considers geometric deformation and local detailed features during feature extraction, enabling the model to more flexibly handle complex marine scenarios while reducing the number of parameters.

### Experimental results on complex maritime scenes

**Fig. 9.**
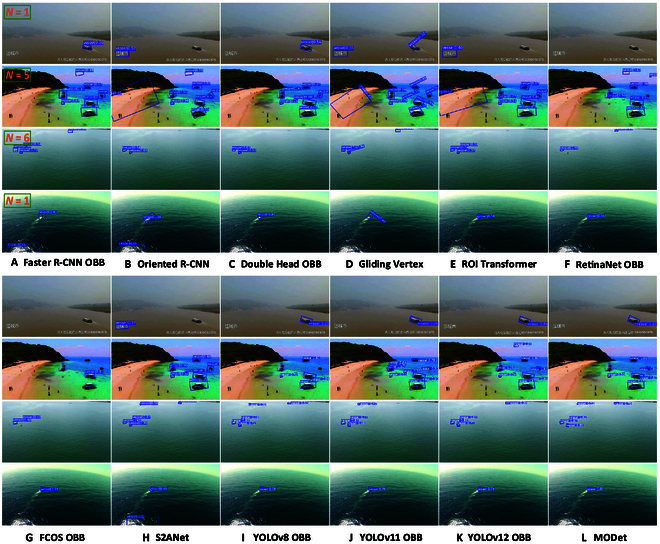
Visual presentation of 12 oriented detection methods on the MS2ShipOBB dataset. Four distinctive images from the MS2ShipOBB dataset are selected as demonstration examples. From the first row to the fourth row, there are inland, shoreline, nearshore, and offshore scenarios, respectively. (A) to (L) show the experimental results of 12 methods, i.e., (A) Faster R-CNN OBB, (B) Oriented R-CNN, (C) Double Head OBB, (D) Gliding Vertex, (E) ROI Transformer, (F) RetinaNet OBB, (G) FCOS OBB, (H) S2ANet, (I) YOLOv8 OBB, (J) YOLOv11 OBB, (K) YOLOv12 OBB, and (L) MODet. The total number of annotated targets is displayed in the top-left corner of the first column.

**Fig. 10.**
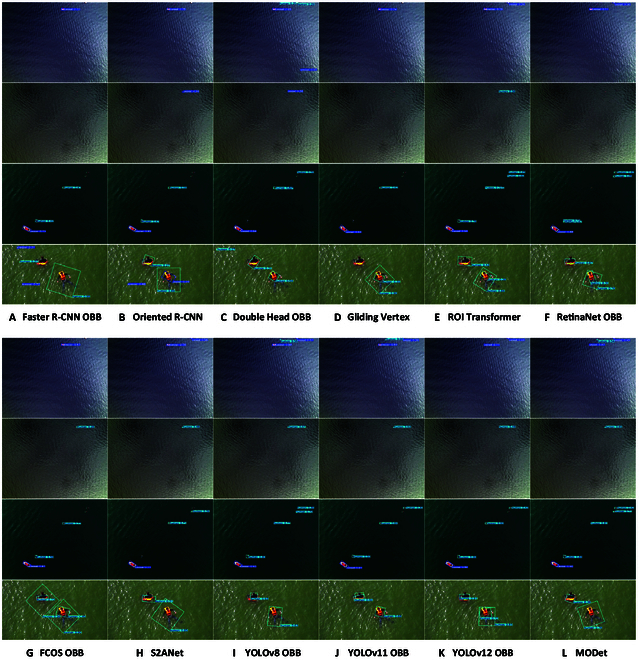
Visual presentation of 12 oriented detection methods on the SeaDronesSeeOBB dataset, including 3 classes, i.e., vessel, human, and obstacle. From the first row to the fourth row, there are 4 scenes, respectively. (A) to (L) show the experimental results of 12 methods, i.e., (A) Faster R-CNN OBB, (B) Oriented R-CNN, (C) Double Head OBB, (D) Gliding Vertex, (E) ROI Transformer, (F) RetinaNet OBB, (G) FCOS OBB, (H) S2ANet, (I) YOLOv8 OBB, (J) YOLOv11 OBB, (K) YOLOv12 OBB, and (L) MODet.

MODet demonstrates excellent performance in both inland and marine scenarios. To further validate its effectiveness across diverse and complex maritime environments, we conducted experimental analysis on all 12 models using the MS2ShipOBB dataset. MS2ShipOBB encompasses hazy inland waterways, complex shoreline scenarios, nearshore scenarios with small targets, and offshore scenarios with glare. The visual detection results presented in Fig. 9 further illustrate how MODet accurately detects the correct number of vessels. In comparison, Faster R-CNN OBB [24] and Oriented R-CNN [38], with their weaker feature extraction capabilities, exhibit the highest false detection rates in complex maritime scenes and often misdetect cloud shapes in the sky and sun glare on sea surface as vessels. Although Gliding Vertex [25], Double Head OBB [23], and RoI Transformer [22] show reduced false detection rates, their detection accuracy remains inferior to one-stage methods. Apparently, Double Head OBB [23] fails to learn small-target features, resulting in the poorest performance for detecting small targets. While Gliding Vertex [25] provides correct detections, its Gliding Mechanism leads to suboptimal alignment between the anchor box and the target. By contrast, RoI Transformer [22], which incorporates attention mechanisms for targets and delivers satisfactory results, still experiences false detections and suffers from slow inference speeds. RetinaNet OBB [28] and FCOS OBB [27] show limited effectiveness in complex scenarios, especially in hazy inland waterway environments where learning vessel features is challenging. Their performance on small targets is also obviously underperforming. S2ANet [26] performs better in detecting small targets and in hazy inland waterway scenes but is easily confused by glare. YOLOv8 [8] and YOLOv11 [29], with stronger feature learning capabilities than the previous 8 oriented detection methods, exhibit outstanding performance across various maritime scenarios, including hazy inland waterways, complex shorelines, small targets, and the glare on sea surface. Although their performance is exceptional, they rely on standard convolutional kernels. Compared to MODet, which employs LDConv [39], their detection accuracy is slightly lower. Furthermore, YOLOv12 [30] performs better in simpler scenarios (e.g., inland and nearshore), even achieving higher detection accuracy than MODet. However, when facing complex shoreline, offshore scenes and vessels with wakes, its generalization ability is inferior to that of our MODet. Furthermore, Fig. 10 also highlights its limitations in complex scenarios; i.e., when humans and obstacles are overly abundant, its detection performance lags behind that of YOLOv11 [29] and YOLOv8 [8]. Nevertheless, its effectiveness remains competitive compared to other 2-stage approaches. Although 2-stage methods generally suffer from slower inference speed, their capacity for feature learning should not be underestimated, as methods like ROI Transformer [22] and Double Head OBB [23] sometimes even outperform S2ANet [26] and FCOS OBB [27].

### Ablation study

To further validate the effectiveness of MODet, we carried out an ablation study following the completion of our experiments. Given the diverse scenarios and target variations in the MS2ShipOBB dataset, we chose it for validation. As shown in Table 4, we evaluated the performance of LDFusion, Neck with LDConv, and the shared detection head in MODet using 3 metrics, i.e., mAP@0.5–0.95, parameters, and giga floating-point operations per second (GFLOPs). The experimental setup and other training parameters were consistent with Results and Discussion. Model n-n-n represents the original YOLOv8 [8]. Model B-n-n incorporates the LDFusion module into the backbone for feature extraction and multi-scale fusion. Obviously, the consideration of LDFusion substantially improves accuracy while reducing the parameters. Model n-N-n replaces the standard convolution in the neck with LDConv, resulting in a modest improvement in detection accuracy. The comparison between Model B-n-n and Model n-N-n highlights that LDConv surpasses standard convolutions in feature extraction, particularly in terms of accuracy, which benefits from its randomized initial position and irregular convolutional kernel. Model n-n-H demonstrates the serious impact of the shared head in lightweight models. The comparison between Model n-n-H and Model B-N-n shows that LDFusion achieves the best performance in feature extraction, while the share head excels in reducing the parameters. Although the Neck with LDConv achieves some improvements in both aspects, its gains are relatively modest. Ultimately, combining all 3 components results in the highest detection accuracy and the smallest parameters.

**Table 4.** The mAP and model size of 8 different models on 3 maritime datasets. The symbol “✓” indicates that the module is included in the model, whereas the symbol “✗” denotes that the module is not considered. The best-performing results are highlighted in bold.

Number	Backbone-LDFusion	Neck-LDConv	Head-SConvs	mAP@0.5–0.95			Params/M
				InlandVessel	SeagullOBB	MS2ShipOBB	
Model n-n-n	✗	✗	✗	0.827	0.784	0.707	3.08
Model B-n-n	✓	✗	✗	0.852	0.815	0.724	2.89
Model n-N-n	✗	✓	✗	0.844	0.806	0.719	2.97
Model n-n-H	✗	✗	✓	0.838	0.799	0.713	2.38
Model B-N-n	✓	✓	✗	0.879	0.842	0.757	2.72
Model B-n-H	✓	✗	✓	0.863	0.831	0.746	2.13
Model n-N-H	✗	✓	✓	0.859	0.829	0.737	2.26
Model B-N-H	✓	✓	✓	**0.897**	**0.872**	**0.798**	**2.02**

### Discussion

Although MODet demonstrates meaningful improvements in multi-scale and oriented object detection from the perspective of UAV-based maritime applications, certain limitations remain. Its ability to learn multi-scale features enables it to effectively capture small vessels in complex maritime environments. However, it occasionally suffers from classification confusion, such as misidentifying navigation buoys as ships. It suggests that relying solely on visual information may not be sufficient for reliable target recognition in cluttered scenes. To address this issue, we plan to integrate auxiliary sensors like radar and AIS into the UAV system for cooperative detection. The fusion of visual and nonvisual modalities is expected to enhance detection robustness and reduce false positives in ambiguous cases. Moreover, although the inference speed of MODet has been substantially improved, there is still room for further optimization. In future work, we intend to perform model pruning by eliminating redundant neurons and convolutional kernels to reduce the computational complexity. This strategy is particularly important for deployment on resource-constrained platforms (e.g., Jetson Nano and Xavier). We will conduct extensive experiments on these embedded systems to verify the practical feasibility of our approach. Finally, to ensure its generalizability and applicability in real-world maritime scenarios, we plan to expand our dataset to include challenging conditions (i.e., adverse weather, poor visibility, and congested port environments). It will allow MODet to be better adapted to diverse maritime surveillance needs and further enhance its operational value in UAV-based maritime surveillance systems.

## Conclusion

In this work, we propose a multi-scale lightweight model for oriented detection from a UAV perspective. We replace horizontal box detection with oriented box detection, which is beneficial for determining the direction of targets and tracking them from the UAV. MODet first uses the irregular convolution of LDFusion in the backbone to extract maritime scene features and utilizes residual connections to reach shallow-to-deep feature fusion. Additionally, we further reduce the parameters by constructing an oriented detection head with SConvs, alleviating the computational burden on edge devices. Furthermore, we collected and annotated a new inland water dataset, and then re-annotated the existing Seagull and MS2Ship datasets to serve OBB detection. We finally evaluate the performance of MODet in real inland, marine, and complex maritime scenes. MODet is compared with 11 different oriented detection methods, and the results indicate that, compared to the latest method, although MODet achieves only a 3.27% improvement in accuracy, it reduces the number of parameters by 24.40%, demonstrating a markedly enhancement in model efficiency without compromising performance.

## Methods

**Fig. 11.**
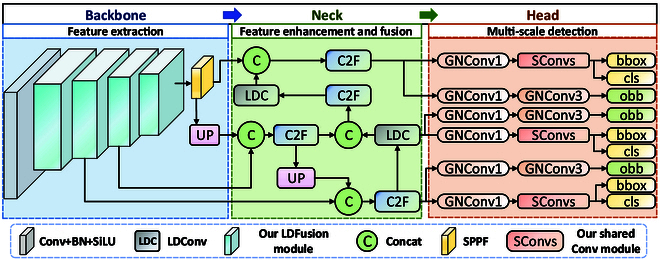
The architecture of our MODet. The entire framework consists of 3 parts, i.e., the backbone with multi-scale feature extraction, the neck with LDConv [39], and the oriented detection head. In the backbone, the LDFusion with LDConv [39] is constructed to achieve multi-scale feature extraction. The PAN-FPN [44] with LDConv [39] is exploited in the neck to improve small-target detection. The oriented detection head with shared convolution reduces model parameters and improves inference speed.

For the task of oriented vessel detection in UAV imagery, we propose a lightweight OBB model (as shown in Fig. 11). Considering the dynamics and unpredictability of UAV-based visual surveillance systems, vessels captured from a UAV perspective often appear at various angles and altitudes. Compared to HBB, OBB is clearly more suitable for maritime surveillance. However, maritime UAVs are often tasked with more than one mission (e.g., search and rescue). To fulfill these roles, UAVs must carry multiple mission-specific devices, which severely limit the available onboard computational resources. As a result, large-scale models are impractical for deployment on such platforms. To address this constraint, we propose a shared convolutional detection head for OBB, aiming to meet the lightweight detection requirements of maritime UAVs. However, lightweight models often suffer from reduced detection accuracy. To ensure accuracy while maintaining efficiency, we provide LDFusion, which randomly integrates multi-scale features and fully accounts for vessel details at different scales, thereby enhancing the detection and recognition capabilities. The pseudocode of the proposed method is shown in Algorithm 1, which mainly includes the backbone with multi-scale feature fusion with LDFusion, the neck with lightweight feature fusion with LDConv [39], and the oriented head with shared convolution.


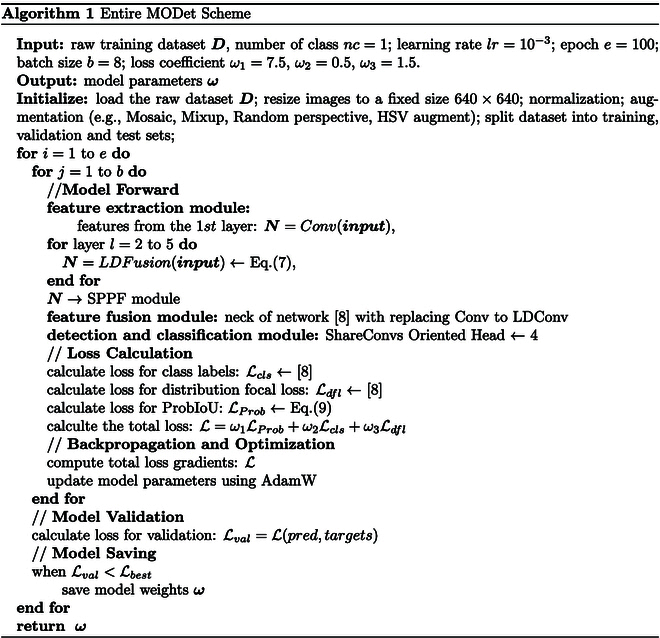


### Multi-scale feature extraction and fusion

The backbone of the network is important for extracting image features. CSPNet [40] allows the separation and merging of feature maps to reduce redundant computations and optimize gradient flow, improving computational efficiency and learning capacity. More detailed feature information is needed in complex maritime environments. Meanwhile, UAVs as edge devices require lightweight network models. Therefore, we choose CSPNet [40] as the feature extraction network for maritime scenarios. In the backbone network, convolution operations demonstrate strong feature extraction capabilities. However, there are 2 limitations in the current convolution. Firstly, convolution operations are limited to a local window, making them unable to capture information from other locations. Secondly, the convolution kernel is fixed to a square size of k×k, with the number of parameters increasing quadratically. Therefore, to efficiently extract maritime features from a UAV perspective, we introduce an LDConv [39] layer to generate different initial sampling positions for convolution kernels with any size, and then create the LDFusion module to extract and fuse different-scale features. Additionally, the parameter growth trend of LDFusion is linear, reducing the computational pressure on UAVs. We present the derivation process for the proposed LDFusion module.

#### LDFusion module

**Fig. 12.**
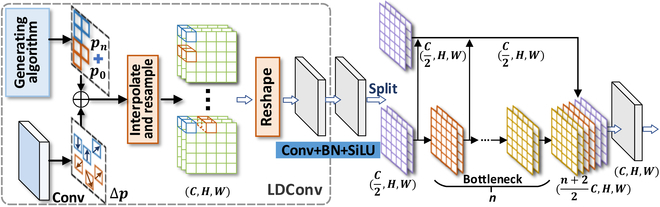
The architecture of our LDFusion. It is designed to make the backbone lightweight while enabling multi-scale feature extraction. Firstly, LDFusion enhances multi-scale feature extraction and stability by introducing irregular convolution (LDConv) and the random initialization position algorithm, respectively. Then, residual connections are repeatedly employed to fuse shallow and deep features, further ensuring effective multi-scale feature learning.

The LDConv [39] enhances the capability of convolution kernels to capture geometric information in local areas by learning dynamic sampling positions. Subsequently, the features are split and residual connections are repeatedly employed to fuse shallow and deep features. Therefore, it enables cross-scale feature fusion, effectively combining local details with global contextual information. By integrating LDConv [39] and C2F [8], we achieve a more precise feature representation under different scales and angles, as illustrated in Fig. 12. LDFusion preserves fine details while expanding the receptive field of the backbone, enhancing the ability to understand targets and improving detection accuracy. The position-aware characteristics of LDConv [39] combined with the efficient computational methods of C2F [8] improve the flexible handling of feature maps while avoiding redundant computations. This is particularly crucial for real-time object detection tasks on edge devices like UAVs. Moreover, the offset convolution in LDConv [39] allows for flexible adjustment of sampling positions, making the module more adaptable to object deformations. C2F [8] ensures that these local geometric features can be further fused and reinforced across multiple scales, thereby enhancing the robustness of detection against variations in object shapes. When the input feature $X_{\text{out}}$ undergoes multi-scale feature extraction and cross-scale fusion through the LDFusion module, the output feature $X_{\text{Fusion}}$ is obtained by

$$Y=\text{Conv}\left(\text{LDConv}\left(X_{\text{out}}\right)\right).$$ (5)

**Fig. 13.**
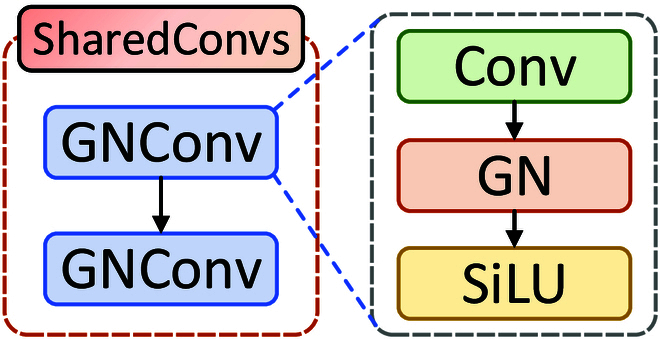
The architecture of our SConvs. It consists of 2 GNConv layers. GN in the architecture is group normalization.

Split Y along the channel dimension to obtain 2 subsets $Y_{1}$ and $Y_{2}$. Perform a Bottleneck operation on one of the subsets to obtain the feature $Y_{B}$

$$Y_{B}=\left[B_{1}\left(Y_{2}\right),\;B_{2}\left(Y_{2}\right),\ldots ,\;B_{n}\left(Y_{2}\right)\right],$$ (6)

where $B_{i}$ represents the *i*th Bottleneck module. Finally, the split $Y_{1}$ and the outputs from all Bottleneck modules are fused to obtain the final output feature as follows:

$$X_{\text{Fusion}}=\text{Conv}\left(\text{Concat}\left(Y_{1},\;Y_{B}\right)\right).$$ (7)

### Lightweight oriented detection

It is important for resource-constrained edge devices to design a flexible oriented detection head to address the dynamics and diversity on maritime targets. Compared to traditional detection methods, the shared-convolution oriented detection head can better handle the detection of targets with angles, enhancing both localization precision and classification performance. Therefore, considering the limited resources of UAVs and the multiple direction of maritime targets, we propose an oriented detection head with shared convolution. It effectively reduces the number of parameters through SConvs, ensuring a lightweight model that is more suitable for resource-constrained UAVs. Moreover, it can flexibly adapt to the rotation, deformation, and scale variations of targets. It resolves the challenges posed by differing target orientations, eventually providing a more efficient solution for maritime safety surveillance.

As a vital part of the SConvs module (as shown in Fig. 13), the ConvGN module is primarily motivated by the advantages of the group normalization (GN) mechanism. Compared to batch normalization (BN) [41], GN [42] maintains training stability when the mini-batch size is small or unbalanced. GN [42] normalizes the mean and variance of each feature channel, reducing variations in input distribution and alleviating internal covariate shift. Moreover, GN [42] is more flexible with input images of different sizes because it does not rely on all samples in the batch. Applying convolutional operations before GN [42] is advantageous since convolutional layers can learn more complex feature representations. It not only reduces the dimensionality of the feature maps and alleviates computational burden but also extracts higher-level features, enhancing generalization. Therefore, we introduce the ConvGN module based on GN [42]. For the input feature Z, after the ConvGN operation, the output feature is given by

$$Z_{\text{out}}=\text{SiLU}\left(\mathrm{GN}\left(\text{Conv}\left(Z\right)\right)\right),$$ (8)

with SiLU and GN being the activation function and group normalization, respectively.

### Loss function

Assessing the degree of overlap between detected boxes and ground-truth boxes is crucial for evaluating model performance in detection. The IoU only provides a discrete value and fails to reflect the probability distribution of the overlap. Therefore, we utilize ProbIoU [43] to model the overlapping area between prediction boxes and ground-truth boxes. ProbIoU [43] represents the IoU between OBBs by measuring the distance between 2-dimensional Gaussian distributions, resulting in a probability distribution instead of a single IoU value. The probability distribution can comprehensively describe the overlap between prediction and ground-truth boxes, providing richer information. The difference $L_{\text{Prob}}$ between the rotated target box and the ground-truth box is expressed by

$$L_{\text{Prob}}=1-\sqrt{1-\int_{{\mathbb{R}}^{2}}\sqrt{p\left(x\right)q\left(x\right)}\mathrm{dx}},$$ (9)

where *p*(*x*) and *q*(*x*) represent the predicted and ground-truth Gaussian target boxes, respectively, following a 2-dimensional Gaussian distribution Ω. The distribution is defined as

$$\Omega ∼\left\{\begin{array}{cc} \mu & =\left[x_{0},\;y_{0}\right],\\ C & =\left[\begin{array}{cc} a{\text{cos}}^{2}\theta +b{\text{sin}}^{2}\theta & \frac{1}{2}\left(a-b\right)\text{sin}\left(2\theta \right)\\ \frac{1}{2}\left(a-b\right)\text{sin}\left(2\theta \right) & a{\text{sin}}^{2}\theta +b{\text{cos}}^{2}\theta \end{array}\right] \end{array}\right.$$ (10)

where μ and C are the mean and covariance matrix of the Gaussian distribution Ω, respectively. *a*, *b*, and θ represent the semi-major axis, semi-minor axis, and rotation angle of the enclosing ellipse for the target, respectively. The similarity measure ProbIoU, based on the Hellinger distance, is derived from [43]. It allows for a more accurate evaluation of detection methods and provides additional reference information during the optimization process.

## Data Availability

The source code and datasets are available at https://github.com/huangyanh/MODet.
